# Full-Line Idler Fault Monitoring in Belt Conveyors via UWFBG-DAS and Characteristic Energy Feature Analysis

**DOI:** 10.3390/s26154905

**Published:** 2026-08-03

**Authors:** Yuyan Liu, Kai Jiang, Chenyang He, Jinxing Qiu, Jiaqi Wang, Xin Gui, Yiming Wang

**Affiliations:** 1 School of International Education, Wuhan University of Technology, Wuhan 430062, China; liu3332@whut.edu.cn; 2 Hubei Longzhong Laboratory, Wuhan University of Technology, Xiangyang Demonstration Zone, Xiangyang 441000, China; 3 School of Information Engineering, Wuhan University of Technology, Wuhan 430062, China; 4 National Engineering Research Center of Fiber Optic Sensing Technology and Networks, Wuhan University of Technology, Wuhan 430062, China

**Keywords:** ultra-weak fiber Bragg grating, distributed acoustic sensing, variational mode decomposition, idler fault monitoring, characteristic energy features

## Abstract

Reliable full-line monitoring of belt-conveyor idlers remains challenging because large numbers of idlers operate under spatially varying structural stiffness and strong industrial vibration. This study develops an ultra-weak fiber Bragg grating distributed acoustic sensing (UWFBG-DAS) method combined with characteristic energy feature analysis for long-distance idler monitoring. The method makes three main contributions. First, a simplified finite-element model identifies the middle crossbeam as an effective vibration-transmission path and guides the deployment of the sensing array. Second, envelope demodulation and variational mode decomposition (VMD) are employed to isolate the fault-sensitive IMF2 component, whose energy is temporally accumulated and evaluated using a zone-specific self-referencing threshold derived from normal-operation data. Third, the method is validated through field deployment and fault-type classification. Approximately 1.2 km of a sensing cable was deployed in a coal-fired power plant, and identifiable characteristic-energy increases were observed in 9 of 10 idler-replacement tests. For three representative fault types, stratified five-fold cross-validation of 300 samples achieved an overall classification accuracy of 90.3%, with a 95% Wilson confidence interval of 86.5–93.2%. These results demonstrate the feasibility of UWFBG-DAS combined with zone-specific characteristic energy analysis for long-distance idler monitoring under spatially heterogeneous industrial conditions.

## 1. Introduction

Belt conveyors have become the backbone of bulk material handling across diverse industrial sectors, including harbor logistics, mining, and coal-fired power plants. Their ubiquity is driven by their structural simplicity, long conveying capabilities, continuous operation, and low maintenance costs [1]. In large-scale energy production systems, particularly coal-fired power plants, belt conveyors perform the critical task of continuous fuel transport. Consequently, their operational status directly dictates the safe and stable operation of the generation units, as well as overall production efficiency [2]. A typical belt conveyor relies on an extensive network of idlers as its primary load-bearing framework. Due to their vast quantity and the high-load, continuous operating conditions typical of coal-fired power plants, idlers are highly susceptible to degradation, accounting for approximately 51% [3] of all conveyor mechanical failures [4]. Specifically, faults such as bearing wear, seizure, or complete failure [5] drastically amplify the friction coefficient between the idler and the moving belt. This excessive friction inevitably induces abnormal local temperature rises and serves as a primary catalyst for catastrophic belt fires [6]. Consequently, effective condition monitoring and early warning of idler faults are of paramount engineering importance and safety value.

Traditionally, idler inspection has predominantly relied on manual walk-through inspections [7]. However, this approach is fundamentally limited by extensive spatial spans, intensive labor requirements, and inherent latency, rendering the timely detection of sudden faults highly improbable. Driven by these limitations, the paradigm of idler health monitoring is rapidly shifting toward automation, precision, and intelligence [8]. In this context, achieving continuous, full-line spatial awareness of the conveyor’s structural condition has emerged as the cornerstone for developing next-generation intelligent monitoring systems. To achieve such intelligent monitoring, two predominant sensing paradigms are currently employed: discrete electronic sensors and distributed optical fiber sensors. The former typically relies on point-based instruments deployed at specific nodes, comprising accelerometers [9], microphones [10], acoustic emission sensors [11], and infrared thermal cameras [12]. However, these discrete approaches yield spatially fragmented data restricted to specific idler nodes. Their limited coverage and the immense complexity of scaling them into extensive networks fundamentally preclude a seamless, full-line perception of the conveyor’s structural state. To overcome these spatial bottlenecks, distributed optical fiber sensing (DOFS) technology [13] has emerged as a transformative alternative, distinguished by its intrinsic immunity to electromagnetic interference and unparalleled long-distance measurement capabilities. Among DOFS variants, Rayleigh scattering-based distributed acoustic sensing (DAS) systems are widely adopted for their continuous dynamic measurement capabilities. Nevertheless, in practical engineering applications, these conventional systems are inherently bottlenecked by low signal-to-noise ratios (SNR) and restricted spatial localization accuracy [14]. Consequently, achieving highly reliable dynamic monitoring within the intensely noisy and vibrationally complex environments of coal-fired power plants remains a formidable challenge.

In recent years, ultra-weak fiber Bragg grating (UWFBG) array-based DAS technology has advanced rapidly. Unlike conventional Rayleigh-scattering DAS, adjacent UWFBGs provide stable reflection boundaries for two-beam interferometry. The low reflectivity of each grating permits thousands of sensing nodes to be multiplexed along a single fiber, improving the repeatability and spatial definition of distributed vibration measurements [15,16]. Nevertheless, coal-handling conveyors remain structurally heterogeneous: support stiffness, reinforcement, installation conditions, and background vibration differ markedly among sections. Consequently, a single global threshold can generate false alarms in high-background zones and missed detections in weak-response zones.

To address these issues, this study develops a zone-specific characteristic-energy method for UWFBG-DAS. VMD is first used to separate the non-stationary envelope signal into band-limited intrinsic mode functions (IMFs), after which the energy of the fault-sensitive mode is accumulated over time and evaluated relative to the normal reference value of the same measurement zone [17,18]. Recent studies have also demonstrated self-referencing demodulation for UWFBG vibration sensing under temperature and background-noise interference [19] and FBG-based extraction of weak bearing-fault features [20], confirming the growing relevance of optical-fiber vibration diagnostics in harsh environments. The contributions of the present work are threefold: (1) finite-element-guided deployment of the UWFBG array on the middle crossbeam; (2) an IMF2-based, zone-specific self-referencing energy criterion with temporal statistical enhancement; and (3) field-scale validation together with stratified five-fold cross-validation for three representative idler fault types.

## 2. Proposed Idler Fault Monitoring Methodology

### 2.1. Sensing Principle of UWFBG-Based DAS

**Figure 1:**
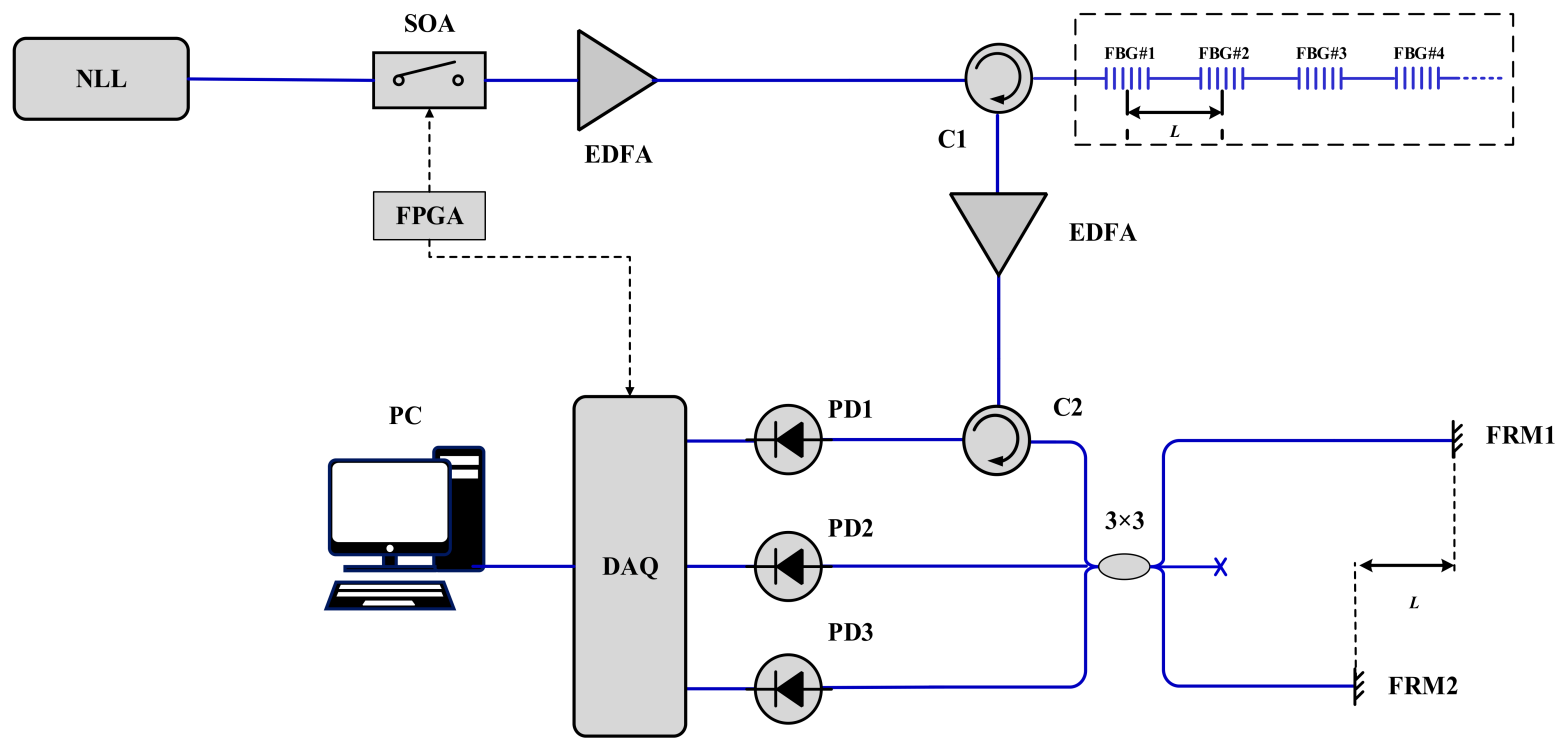
Schematic of the UWFBG-DAS System. Dashed lines indicate control and synchronization connections, and the dashed box denotes the UWFBG array.

The configuration of the proposed UWFBG-based distributed acoustic sensing system is illustrated in Figure 1. The output of a narrow-linewidth laser (NLL) is modulated by a semiconductor optical amplifier (SOA), which is controlled by an FPGA, to generate optical probe pulses. After being amplified by an erbium-doped fiber amplifier (EDFA), the probe pulses are launched into the UWFBG array through optical circulator C1.

The UWFBGs are arranged along the sensing fiber with a uniform spacing L, and each UWFBG reflects a small fraction of the incident optical pulse. Therefore, the reflected pulses from two adjacent UWFBGs contain the differential phase information of the fiber segment between them. The time interval between the two reflected pulses is determined by the UWFBG spacing L and the propagation velocity of light in the fiber.

After returning through C1, the reflected signals are amplified by a second erbium-doped fiber amplifier and subsequently routed by optical circulator C2 into a 3 × 3 unbalanced Michelson interferometer. The interferometer consists of a 3 × 3 optical coupler, a delay fiber, and two Faraday rotator mirrors, denoted as FRM1 and FRM2. The differential delay between the two arms of the interferometer is designed to match the time interval between the reflected pulses from two adjacent UWFBGs.

Consequently, a reflected pulse from one UWFBG propagating through the long arm overlaps with the reflected pulse from the adjacent UWFBG propagating through the short arm. The three coupler outputs can be expressed by Equation (1). The common-mode component is removed by constructing the two orthogonal quantities in Equation (2), and the continuous differential phase is recovered using Equation (3). This three-channel combination reduces sensitivity to optical-power fluctuation and avoids quadrature fading, after which the continuous phase sequence is converted into the vibration waveform.

(1) $$I_{j}(t)=A(t)+B(t)\cos\left[\Delta \varphi (t)+\frac{2\pi (j-1)}{3}\right],\;\;\;j=1,2,3$$

(2) $$X(t)=2I_{1}(t)-I_{2}(t)-I_{3}(t),\;\;\;\;Y(t)=\sqrt{3}\left[I_{2}(t)-I_{3}(t)\right]$$

(3) $$\Delta \varphi (t)=\mathrm{unwrap}\left\{\mathrm{atan}2\left[Y(t),X(t)\right]\right\}$$

When an external vibration acts on the sensing fiber, it changes the local strain and optical path length of the fiber segment between two adjacent UWFBGs. The resulting phase change in the probe light can be expressed as

(4) Δφ=−βLμσ/E

where β is the propagation constant of the optical fiber, L is the spacing between two adjacent UWFBGs, μ is Poisson’s ratio of the fiber, σ is the effective stress acting on the fiber, and E is Young’s modulus of the fiber.

Accordingly, the differential phase variation tracks vibration-induced stress in the fiber segment between adjacent UWFBGs. Temperature variation and cable creep mainly manifest as slowly varying, quasi-static phase components. Before feature extraction, the demodulated phase sequence is therefore mean-removed, components below 1 Hz are suppressed, and zone-specific historical normalization is applied to attenuate slowly varying offsets. The fault-sensitive vibration components investigated in this study are mainly distributed in the tens-of-hertz range; thus, this preprocessing reduces temperature-related low-frequency drift while preserving the dynamic vibration information associated with idler faults.

### 2.2. Finite Element Modeling and Optimal Sensor Deployment

**Figure 2:**
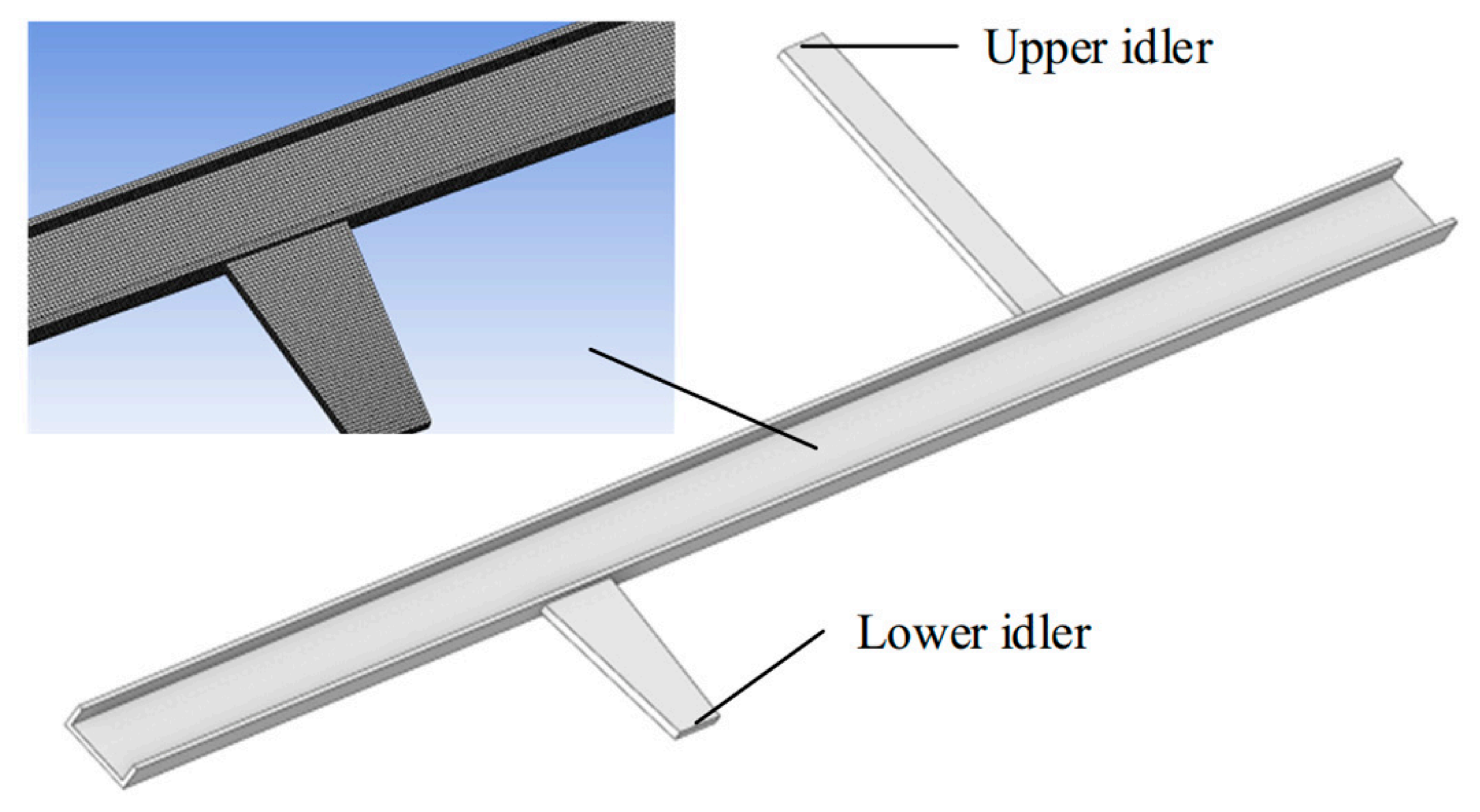
Model Construction Diagram.

Considering the structural complexity of the field conveyor, the finite-element model was simplified while retaining the principal load-bearing and vibration-transmission components. The model consists of the upper idler support, the lower idler support, and the middle crossbeam, with the middle crossbeam taken as the response location. The simplified finite-element model and its principal components are shown in Figure 2. The modeled section is 3000 mm long; the crossbeam is 120 mm wide and 8 mm thick with a grooved cross-section. The upper U-shaped idler support was represented by a 500 × 90 mm rectangular member, and the lower long-idler support by a 6 mm thick trapezoidal member. All dimensions were determined from the field structure. The model was used to analyze the frequency-dependent response of the crossbeam and compare the vibration-transmission characteristics of the upper and lower idler paths, thereby providing a basis for sensor deployment. The belt, detailed bearing contacts, drive drums, and neighboring conveyor sections were not included in the present simplified model.

**Figure 3:**
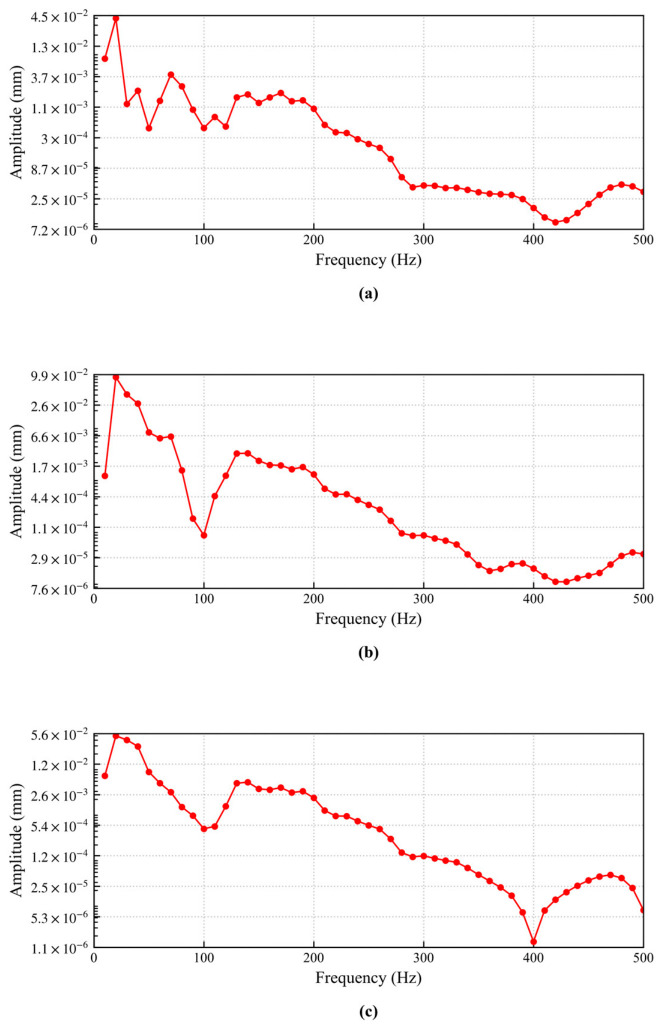
Crossbeam frequency responses of the simplified support structure under (**a**) lower-idler excitation, (**b**) upper-idler excitation, and (**c**) combined upper- and lower-idler excitation.

To examine the frequency-dependent response at the sensing position, a 100 N vertical harmonic load was applied at the lower-idler support, the upper-idler support, and both supports, respectively, while the excitation frequency was swept from 0 to 500 Hz. The response amplitude of the middle crossbeam was extracted under the three excitation conditions, as shown in Figure 3.

Figure 3 shows the frequency responses of the middle crossbeam under the three excitation conditions. Under lower-idler excitation (Figure 3a), the response is relatively strong within 0–100 Hz and exhibits a distinct local peak near 20 Hz, followed by an overall decrease above 200 Hz. Under upper-idler excitation (Figure 3b), a similar low-frequency peak is observed near 20 Hz, and the response gradually weakens with increasing frequency. Under combined excitation (Figure 3c), pronounced response components remain within approximately 20–50 Hz, together with several higher-frequency fluctuations. The three cases consistently indicate that the middle crossbeam is sensitive to low-frequency vibration transmitted from the idler supports.

**Figure 4:**
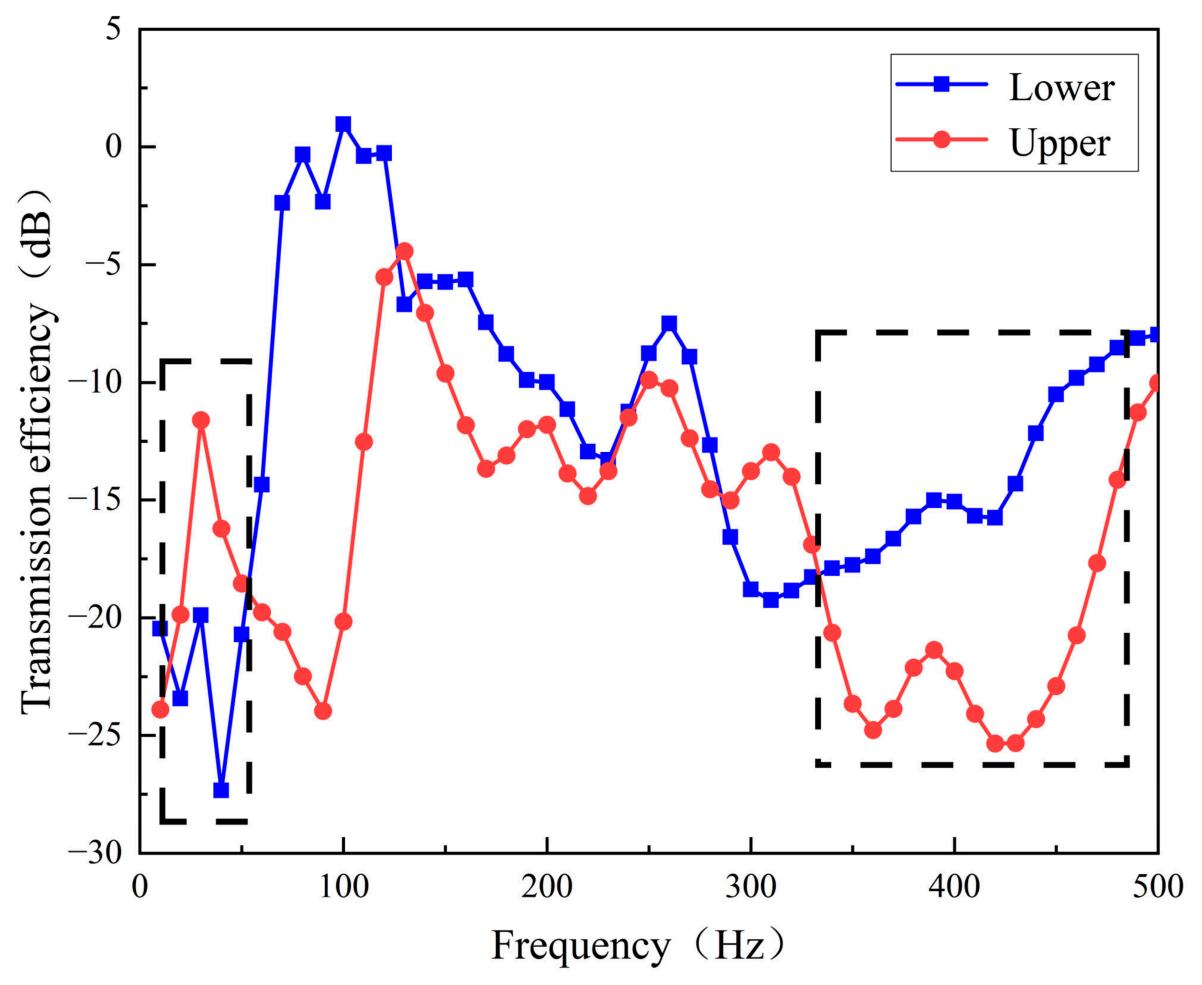
Transmission Efficiency Curves. The dashed boxes highlight representative frequency ranges with pronounced differences between the upper- and lower-idler transmission paths.

Figure 4 compares the vibration-transmission efficiencies of the upper- and lower-idler paths. In the 0–100 Hz range, the two paths exhibit clear differences: the upper-idler path is stronger at the beginning of the sweep, whereas the lower-idler path increases with frequency and becomes dominant above approximately 50 Hz. The two curves show similar trends between 100 and 300 Hz, and the lower-idler path generally maintains higher transmission efficiency above 350 Hz. The local peaks and valleys reflect the frequency-dependent dynamic response of the support structure. Together with the response curves in Figure 3, these results show that vibration from both upper and lower idlers can be transmitted to the middle crossbeam, supporting the placement of the UWFBG array at this position. The low-frequency response observed in the simulation also provides a reference for the subsequent measured-signal analysis.

**Figure 5:**
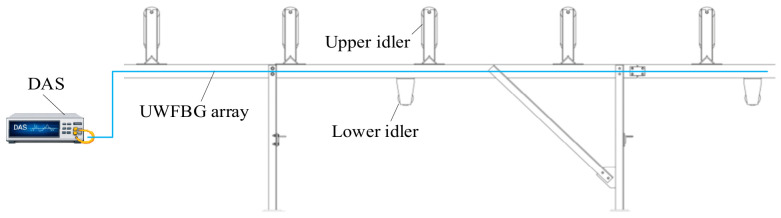
Schematic Diagram of Layout.

To achieve unmanned and intelligent monitoring of idler faults on coal conveying belt conveyors in thermal power plants, and based on preliminary field surveys combined with feasibility analysis of engineering construction, the scheme shown in Figure 5 is adopted as follows: an array of UWFBG is attached along the inner side of the belt conveyor frame crossbeam using 3M adhesive tape, and a layer of epoxy resin is applied on both sides of the optical cable to prevent subsequent detachment. Through a single optical cable, real-time monitoring of all idlers across six belt conveyor sections is realized. At the joints, the optical cables of each section are fusion-spliced and protected by splice closures. Finally, the tail end of the optical cable is connected to a data acquisition instrument for real-time vibration signal acquisition and analysis.

The sensing cable used in the experiments had a nominal center wavelength of 1550.5 nm, a UWFBG reflectivity of 0.004%, and a uniform grating spacing of 3 m. The UWFBG-DAS demodulator supported four-channel synchronous acquisition, a spatial resolution of 3 m, a maximum monitored fiber length of 4 km, and a sampling frequency of 1 kHz. The field deployment used approximately 1.2 km of sensing cable and 420 measurement zones. Previous characterization of a related FBG-array DAS platform developed by our group reported a measurement dynamic range of 97.14 dB at 1 kHz and a phase-noise power spectral density of approximately −80.68 dB re rad/√Hz at 1 kHz, corresponding to a strain resolution of 2.04 pε/√Hz [21].

### 2.3. Adaptive Decomposition of Non-Stationary Vibration Signals

Idler faults generally manifest as periodic or quasi-periodic impact excitations. After transmission through the belt conveyor structure, these impacts modulate the amplitude of the structural vibration response and produce non-stationary vibration signals. To reveal the weak modulation components associated with idler faults, envelope demodulation is first performed, followed by variational mode decomposition (VMD) for adaptive frequency-band separation.

Assume that the belt conveyor system is instrumented with *M* sensor-based measurement zones. The raw vibration signal acquired from the *m*th measurement zone is expressed as

(5) $$x_{m}\left(t\right),m=1,2,\ldots ,M$$

Since idler faults usually appear as periodic or quasi-periodic impulses, their principal effect on the measured vibration is the amplitude modulation of the structural response. Therefore, the analytic signal is first constructed using the Hilbert transform:

(6) $$z_{m}\left(t\right)=x_{m}\left(t\right)+jH\left\{x_{m}\left(t\right)\right\}$$

where $H\left\{\cdot \right\}$ denotes the Hilbert transform operator. The instantaneous envelope of the signal is then obtained as

(7) $$e_{m}\left(t\right)=\left|z_{m}\left(t\right)\right|$$

**Figure 6:**
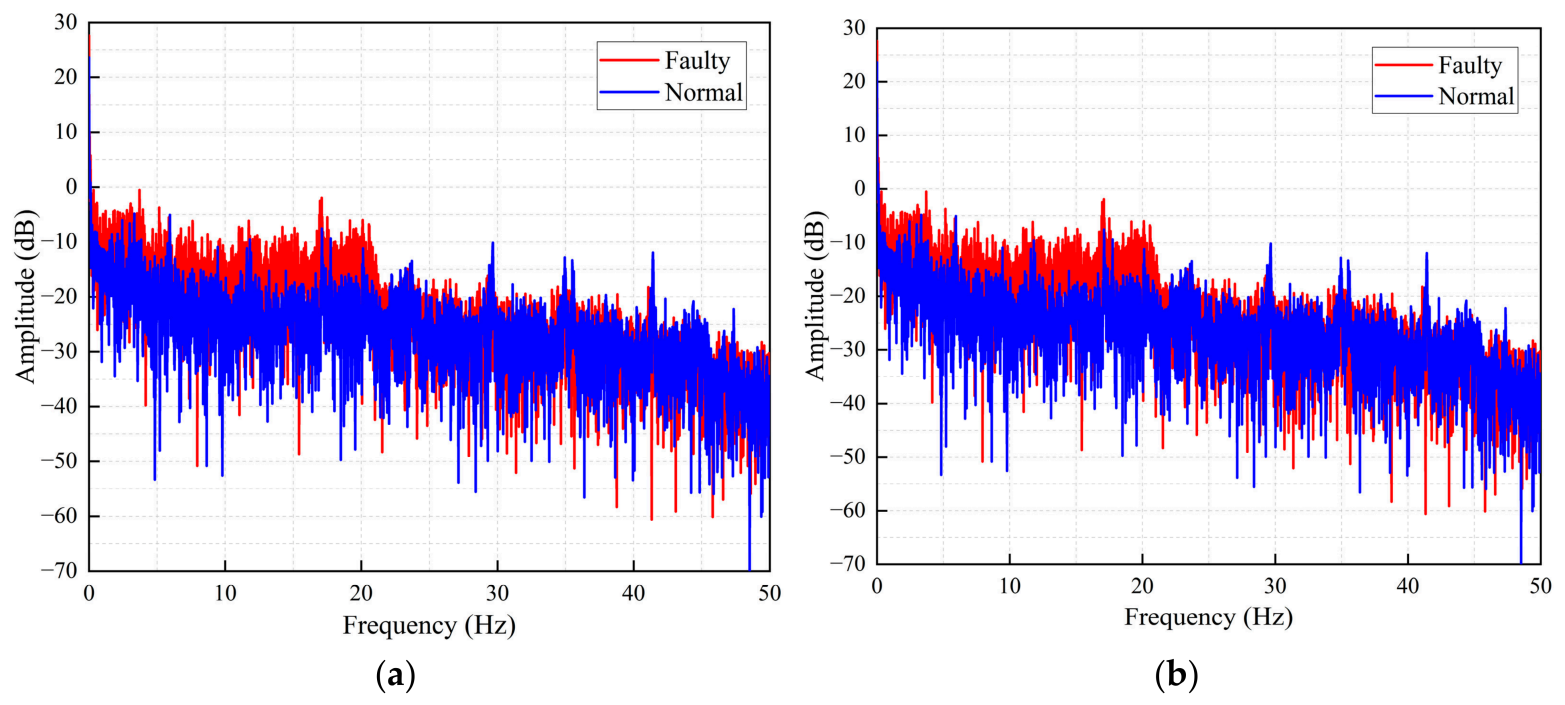
Envelope spectrum analysis. (**a**) Lower idler fault. (**b**) Upper idler fault.

Figure 6 compares the envelope spectra under normal and faulty conditions for the lower and upper idlers. In both cases, the spectral amplitude increases markedly within 0–50 Hz after the faulty idler is installed. The lower-idler fault produces a broad enhancement over the displayed band, whereas the upper-idler fault shows a similarly stable increase. This low-frequency enhancement agrees with the response tendency of the middle crossbeam in Figure 3 and confirms that the UWFBG array can capture the modulation components generated by abnormal idler operation. Because the field spectra contain multiple structural and environmental components rather than a single fixed peak, VMD is subsequently used to separate the signal into characteristic frequency bands.

To further separate the fault-related modulation components from background vibration and structural interference, VMD is introduced. VMD is an adaptive and fully non-recursive signal decomposition method that decomposes a non-stationary signal into a predefined number of band-limited intrinsic mode functions (IMFs), with each mode concentrated around its own center frequency [17,18]. Compared with recursive empirical decomposition methods, VMD offers improved resistance to mode mixing and is therefore suitable for the analysis of complex idler vibration signals.

Although envelope demodulation suppresses the high-frequency carrier vibration and highlights the low-frequency modulation information induced by fault impacts, the envelope signal $e_{m}\left(t\right)$ still contains environmental interference and structural response components. It is therefore decomposed into a sum of *K* band-limited IMFs:

(8) $$e_{m}\left(t\right)=\sum_{k=1}^{K}\mu_{m,k}\left(t\right)$$

The VMD process is formulated as the following constrained variational problem:

(9) ∑k=1K‖∂tδt+jπt∗μm,kte−jωkt‖22μm,k,ωkmin      s.t.∑k=1Kμm,kt=emt

where $\mu_{m,k}\left(t\right)$ and $\omega_{k}$ denote the *k*th mode component and its corresponding center frequency, respectively. In the variational formulation, the analytic signal of each mode is constructed through the Hilbert transform, and its spectrum is shifted to the baseband by frequency demodulation. The total estimated bandwidth of all modes is minimized under the constraint that their sum reconstructs the original envelope signal.

The constrained variational problem is solved iteratively using the alternating direction method of multipliers (ADMM), with the mode components, center frequencies, and Lagrange multiplier updated until convergence. Before fixing the mode number, different K values were compared in preliminary tests in terms of decomposition stability, fault-feature separability, classification accuracy, and computation time. A smaller K produced insufficient separation between adjacent frequency components, whereas a larger K led to mode splitting or redundant components and increased the computational load. Considering both diagnostic accuracy and the processing efficiency required for field-scale multi-zone monitoring, K = 5 was adopted. This setting yielded five well-separated band-limited modes and was used throughout the subsequent analysis.

**Figure 7:**
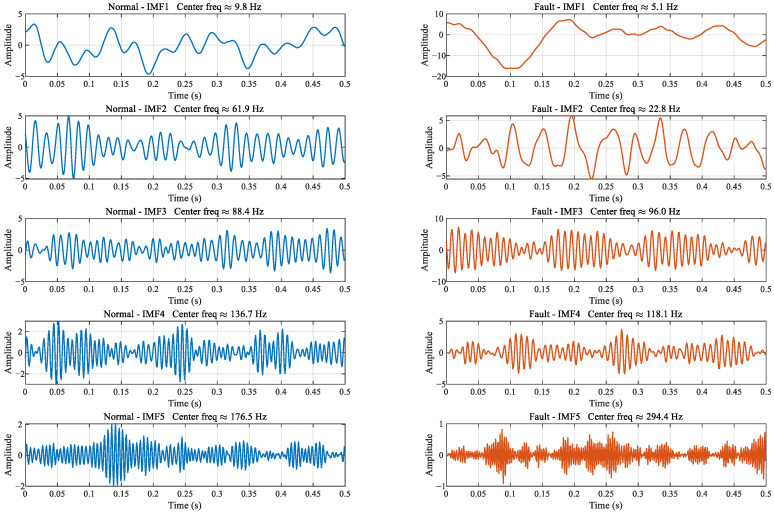
VMD results under normal and faulty conditions.

Figure 7 compares the VMD results before and after replacement with a faulty upper idler. The first five modes exhibit distinct frequency distributions, among which IMF2 shows the most pronounced change. Under the normal condition, IMF2 has a regular, nearly sinusoidal waveform, a center frequency of approximately 61.9 Hz, and an energy proportion of about 24%. Under the faulty condition, its periodicity weakens and irregular impulsive fluctuations appear; the center frequency decreases to approximately 22.8 Hz, while the energy proportion rises to about 45%, corresponding to a relative increase of 87.5%. The center frequency after the fault also falls within the low-frequency response range indicated by Figure 3 and the 0–50 Hz enhancement observed in Figure 6.

IMF1 and IMF3–IMF5 show smaller or less consistent changes in waveform morphology, center frequency, and energy proportion. By comparison, IMF2 presents the clearest combined variation in these three aspects, indicating that the fault mainly redistributes the vibration energy within this frequency band. Therefore, IMF2 was selected as the fault-sensitive mode, and its energy was used to construct the characteristic frequency-band feature.

### 2.4. Construction of Characteristic Energy Features

Based on the envelope demodulation and VMD results obtained in Section 2.3, characteristic energy features are constructed by selecting the fault-sensitive mode and enhancing its persistent fault-related information through temporal statistics. This process maps weak, local vibration anomalies into stable measurement-zone-level feature changes, thereby improving the reliability of idler fault identification under complex operating conditions.

From the decomposed mode set of measurement zone *m*, the component exhibiting the most pronounced and consistent difference between the normal and faulty conditions is selected as the fault-sensitive mode and is denoted as

(10) $$\tilde{\mu_{m}}\left(t\right)$$

Because a mode extracted from a single vibration segment is strongly affected by random operating disturbances, a temporal statistical enhancement strategy is introduced. Assume that *N* mutually independent vibration samples are available. For the *i*th sample segment, the corresponding fault-sensitive mode $\tilde{\mu_{m,i}}\left(t\right)$ is extracted, where i=1,2,…,N. The statistical feature of measurement zone *m* is defined as the time-averaged energy of this mode:

(11) $$E_{m}\;=\;\frac{1}{N}\overset{N}{\underset{i=1}{\sum }}\int_{0}^{T}{\tilde{\mu }}_{m,i}^{2}\left(t\right)dt$$

where *T* is the duration of a single signal segment. By accumulating and averaging multiple vibration segments, random environmental interference is suppressed while persistent fault-related modulation is enhanced. Applying this procedure to all measurement zones yields the statistical feature vector:

(12) $$Ε\;=\;\left[{Ε}_{1},{Ε}_{2},\ldots ,{Ε}_{M}\right]$$

Because the structural form, support stiffness, and environmental conditions vary substantially along the belt conveyor, the normal reference feature level also differs among measurement zones. A uniform threshold is therefore unsuitable for direct horizontal comparison. Instead, each zone is evaluated relative to its own normal reference state. Let the normal reference value of measurement zone *m* be $B_{m}$, which can be estimated from the mean of historical normal data. Let the feature calculated from the latest statistical window be $E_{m}^{new}$. The relative change rate is then defined as

(13) $$R_{m}=\frac{E_{m}^{new}-B_{m}}{B_{m}}$$

When an idler fault occurs within a measurement zone, local impulse modulation is enhanced and the energy of the fault-sensitive mode increases, resulting in a significant rise in $R_{m}$. The fault decision is therefore made according to the following threshold criterion:

(14) $$R_{m}\;>\;\eta_{m\;}\;\Rightarrow \;Fault\;at\;zone\;m$$

where η_m_ is the decision threshold for measurement zone m. For each zone, the normal reference value B_m_ is estimated from the mean characteristic energy of verified normal samples. The corresponding normal relative-change values R_m_ are then calculated using Equation (13), and η_m_ is defined as their 95th percentile. This percentile-based rule accommodates approximately 95% of the historical normal fluctuations while remaining sensitive to persistent energy increases. The threshold is determined independently for every zone and is updated only with verified normal data, thereby avoiding a single global threshold along the conveyor.

## 3. Experiment and Analysis

### 3.1. Synchronous Detection of Multiple Idler Faults

To further evaluate the applicability of the proposed method in real industrial environments, it was applied to the coal conveying belt conveyor system of a thermal power plant in Fujian Province for field verification experiments.

**Figure 8:**
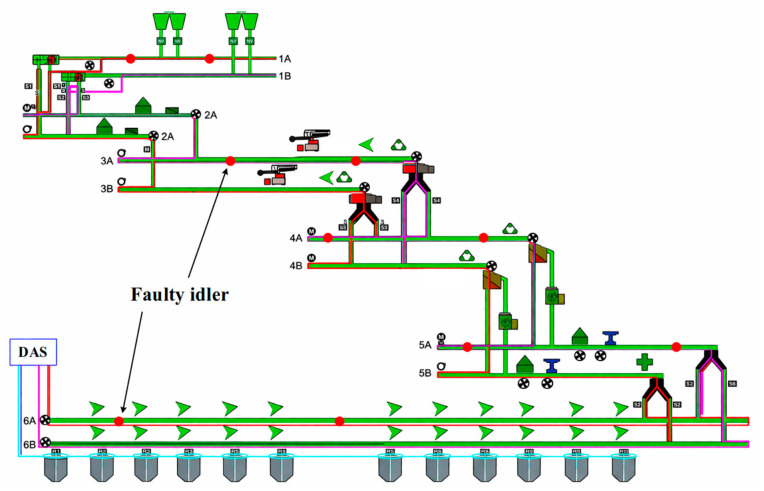
On-site Structural Schematic of the Belt Conveyor.

The coal conveying system of this thermal power plant consists of six belt conveyors connected in series, with a total length of approximately 1.2 km. Its overall structural schematic is shown in Figure 8. Each belt conveyor exhibits significant differences in structural form, support stiffness, and environmental interference, and the background vibration levels and operating conditions also vary among different sections.

To verify the full-line idler fault monitoring capability of the UWFBG array-based vibration acquisition method under complex operating conditions, this study conducted synchronous detection experiments on multiple idler faults without affecting the normal operation of the coal conveying system of the thermal power plant and while ensuring on-site safety. The specific experimental scheme was as follows: five out of the six belt conveyors were selected as experimental subjects. For each of these five conveyors, two representative measurement zones were chosen to carry out idler replacement experiments, resulting in a total of 10 sets of faulty idlers replaced along the entire conveying line to validate the effectiveness of the detection method. It should be noted that, because the second belt conveyor had a relatively short operating time, low equipment usage frequency, a small conveying length, and a limited number of idlers, no replacement experiment was conducted on that section. For the remaining five belt conveyors, the measurement zones selected for idler replacement were located in both structurally reinforced and non-reinforced areas, in order to evaluate the detection performance of the UWFBG array-based vibration monitoring method under different structural stiffness conditions.

**Figure 9:**
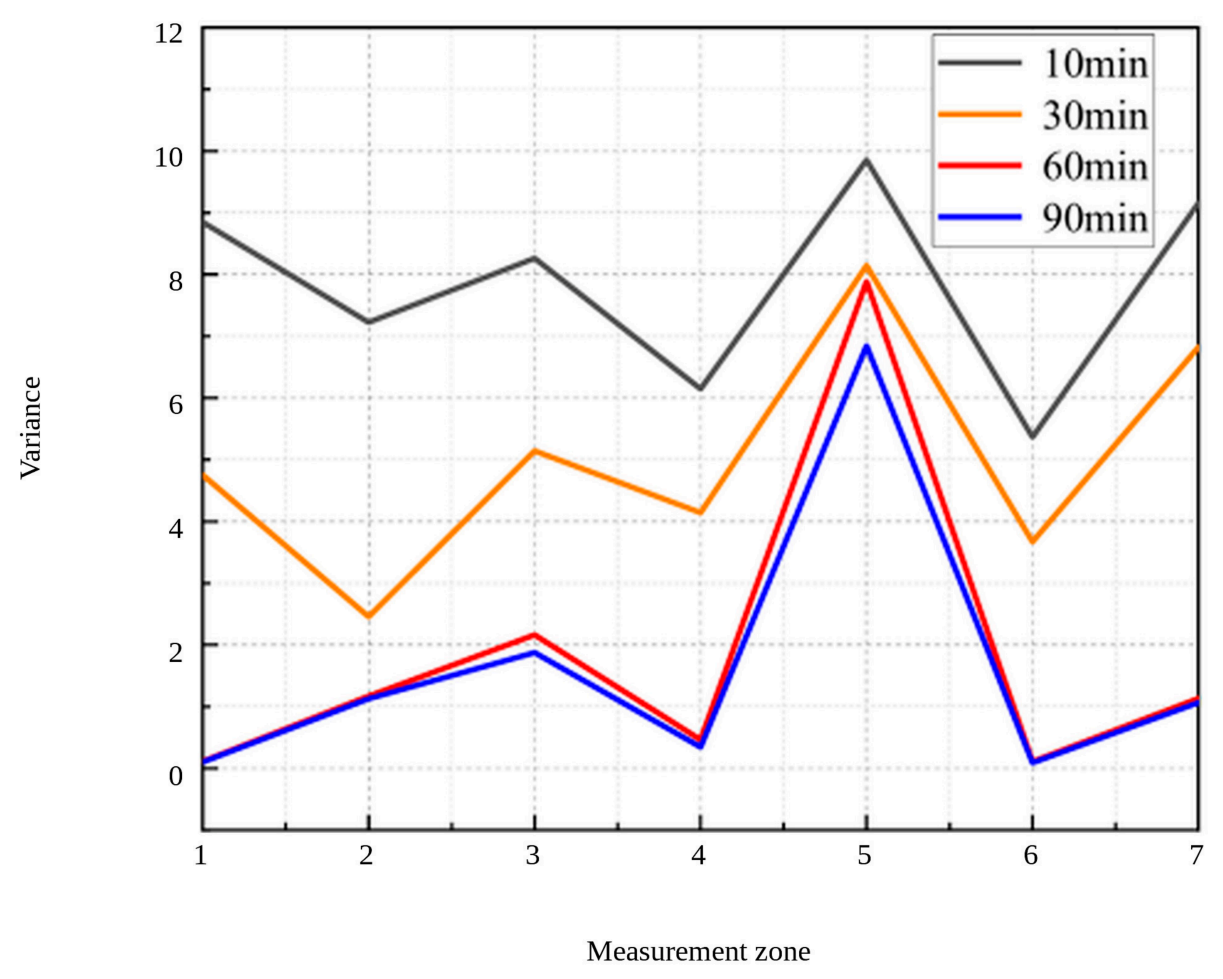
Variance of characteristic-energy statistics obtained using different temporal accumulation windows.

The effect of temporal accumulation length was evaluated using 10-, 30-, 60-, and 90 min windows. Because each elementary signal segment lasted 60 s, these settings corresponded to N = 10, 30, 60, and 90 independent segments in Equation (11). At each window length, the characteristic-energy statistics were calculated for every measurement zone, and the variance among repeated results was used to assess stability. As shown in Figure 9, the 10 min window produces relatively large fluctuations, the variance decreases markedly at 30 min, and the 60 and 90 min results are close. Therefore, 60 min was selected because it provides nearly the same statistical stability as 90 min while reducing the amount of data and computation required for field processing.

The small difference between the 60 and 90 min results indicates that the improvement becomes limited when N exceeds 60. Accordingly, the subsequent field comparisons used 60 min of normal-operation data as the reference and 60 min of faulty-operation data for evaluation.

**Figure 10:**
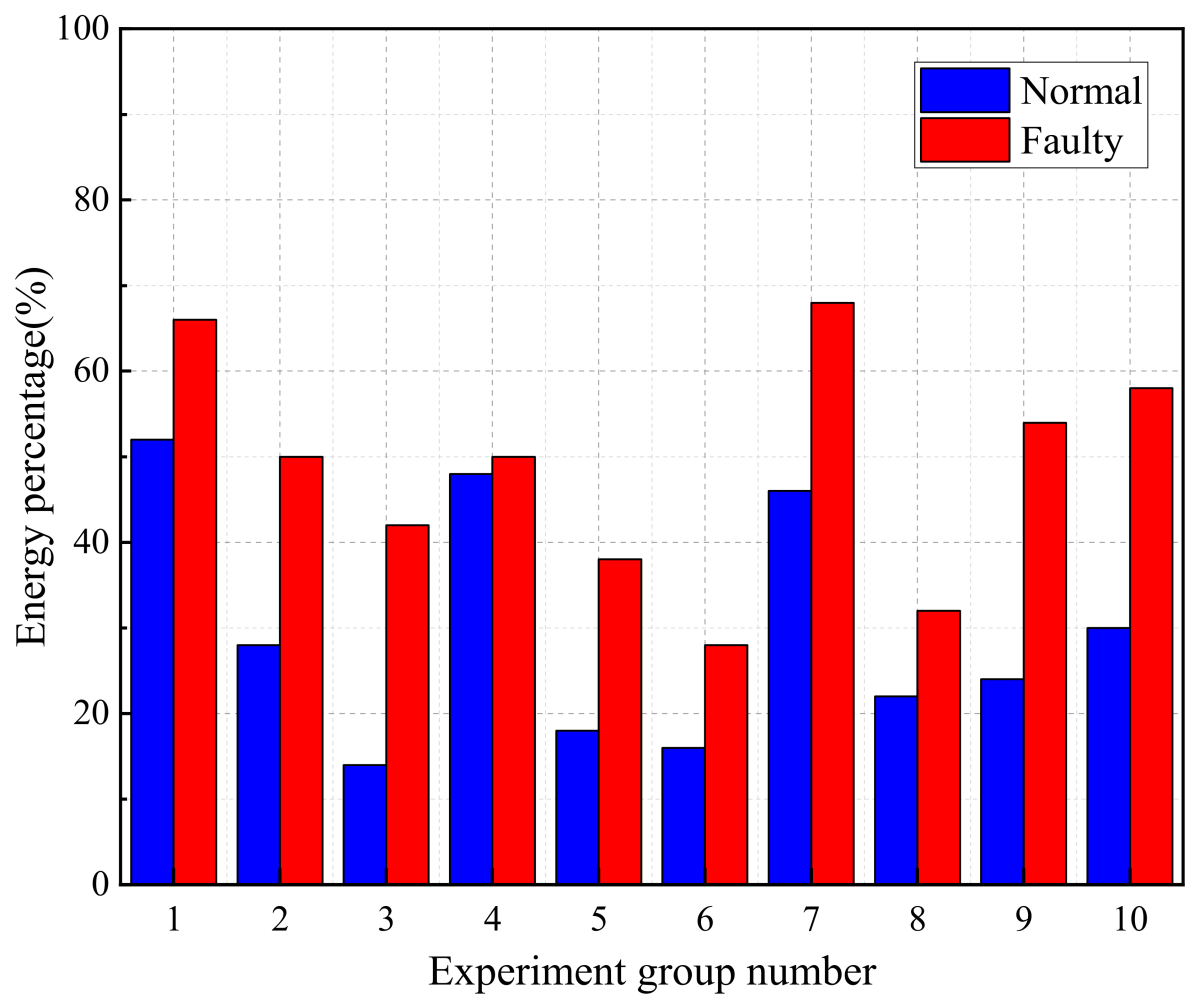
Comparison of Characteristic-Band Energy Proportions under Normal and Faulty Conditions.

After completing the idler replacements, VMD was performed on the vibration signals before and after replacement for each measurement zone, and the variation in the energy proportion of characteristic frequency bands was calculated. The experimental results show that, among the 10 sets of idler replacement experiments conducted, as illustrated in Figure 10, 9 sets exhibited a distinct increase in the energy proportion of characteristic frequency bands after the idler replacement. Specifically, after the faulty idlers were put into operation in the corresponding measurement zones, the low-frequency or mid-frequency characteristic energy of the vibration signal increased significantly compared to the normal state. This phenomenon is consistent with the earlier simulation experiments and fault mechanism analysis results, indicating that the vibration excitation generated by abnormal idler operation can be transmitted through the belt conveyor structure to the beam position and effectively captured by the UWFBG array-based vibration sensing system.

From the experimental results obtained under different environmental interference conditions, it can be observed that the abnormal state after idler replacement can be effectively identified through the change in the energy proportion of characteristic frequency bands, in both structurally reinforced areas and non-reinforced areas. This indicates that the fault identification method based on VMD and energy analysis proposed in this paper, combined with the advantage of zone-specific measurement of the UWFBG array, possesses good applicability under different structural stiffness conditions and can effectively overcome the background vibration variation caused by structural differences in the belt conveyor. For the remaining one experiment in which no significant change in characteristic frequency band energy was observed, an analysis combined with on-site operating conditions suggests the following possible causes: first, strong background vibrations generated by operating equipment near some measurement zones may partially mask the idler vibration signals, reducing the significance of the characteristic change; second, some of the replaced idlers had relatively minor faults, generating weak vibration excitation that is difficult to cause a noticeable change in energy proportion under complex operating conditions. In addition, the vibration transmission path and structural damping characteristics of the belt conveyor structure may also exert certain influences on the propagation of vibration signals.

Overall, in 9 out of the 10 field experiments, the vibration characteristic changes caused by idler replacement were successfully detected, achieving a detection efficiency of 90%. The results demonstrate that, in complex industrial environments, the UWFBG array-based vibration monitoring system can stably identify abnormal idler conditions under different environmental conditions in various belt conveyor sections.

### 3.2. Effective Detection of Different Idler Positions

**Figure 11:**
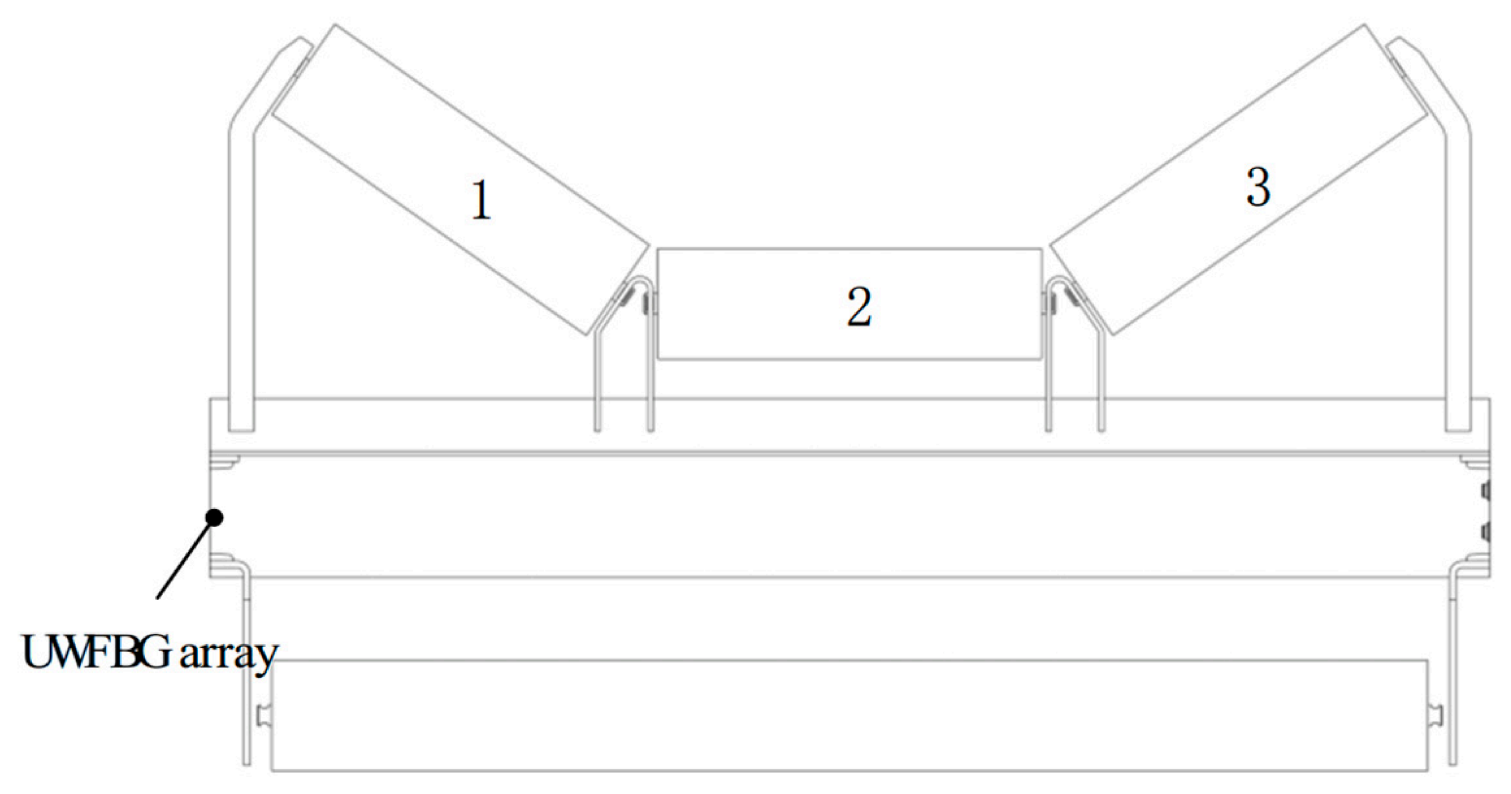
Structural Diagram of the Idler.

In actual engineering deployment, the UWFBG array is installed only on one side of the belt conveyor structure (Figure 11). Therefore, it is necessary to verify the system’s capability to detect idler faults at different positions under this single-side layout. In this section, fault tests were performed by sequentially replacing three idlers at different positions within the same idler group to evaluate the system response. The three idlers are: idler #1 near the UWFBG array, idler #2 at the bottom of the U-shaped group, and idler #3 far from the UWFBG array. The feasibility of single-side detection was verified by comparing frequency-domain features before and after replacement.

**Figure 12:**
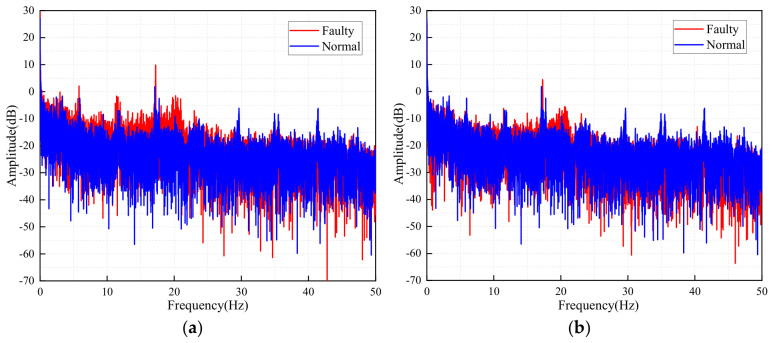
Envelope spectrum analysis of idlers at different positions. (**a**) Idler #2; (**b**) Idler #3.

After replacing idler #1 (Figure 6b), the spectral energy in the low-frequency band (5–25 Hz) increased significantly, and the periodic resonance peaks present during normal operation essentially disappeared after the fault. After replacing idler #2 (Figure 12a), the overall trend was similar to that of idler #1, but with a slightly lower energy increase, indicating that the UWFBG array’s response intensity varies with idler position. After replacing idler #3 at the farthest location (Figure 12b), the spectral change amplitude was clearly reduced, yet a noticeable energy rise was still observed around 20 Hz. This suggests that even for idlers distant from the array, abnormal conditions can still produce characteristic frequency responses that are effectively captured by the UWFBG array.

Summarizing the results of the three idler replacement experiments, when an idler fails, the spectral energy in the 5–25 Hz low-frequency band generally increases, while the periodic resonance features weaken or disappear. As the distance between the idler and the UWFBG array increases, the amplitude of the abnormal response decreases, but distinguishable features remain near specific frequencies. This demonstrates that the single-side UWFBG array layout can effectively sense the operational state changes in the entire idler group and possesses the capability to monitor and identify idler faults along the entire belt conveyor.

### 3.3. Effective Discrimination of Fault Types

**Figure 13:**
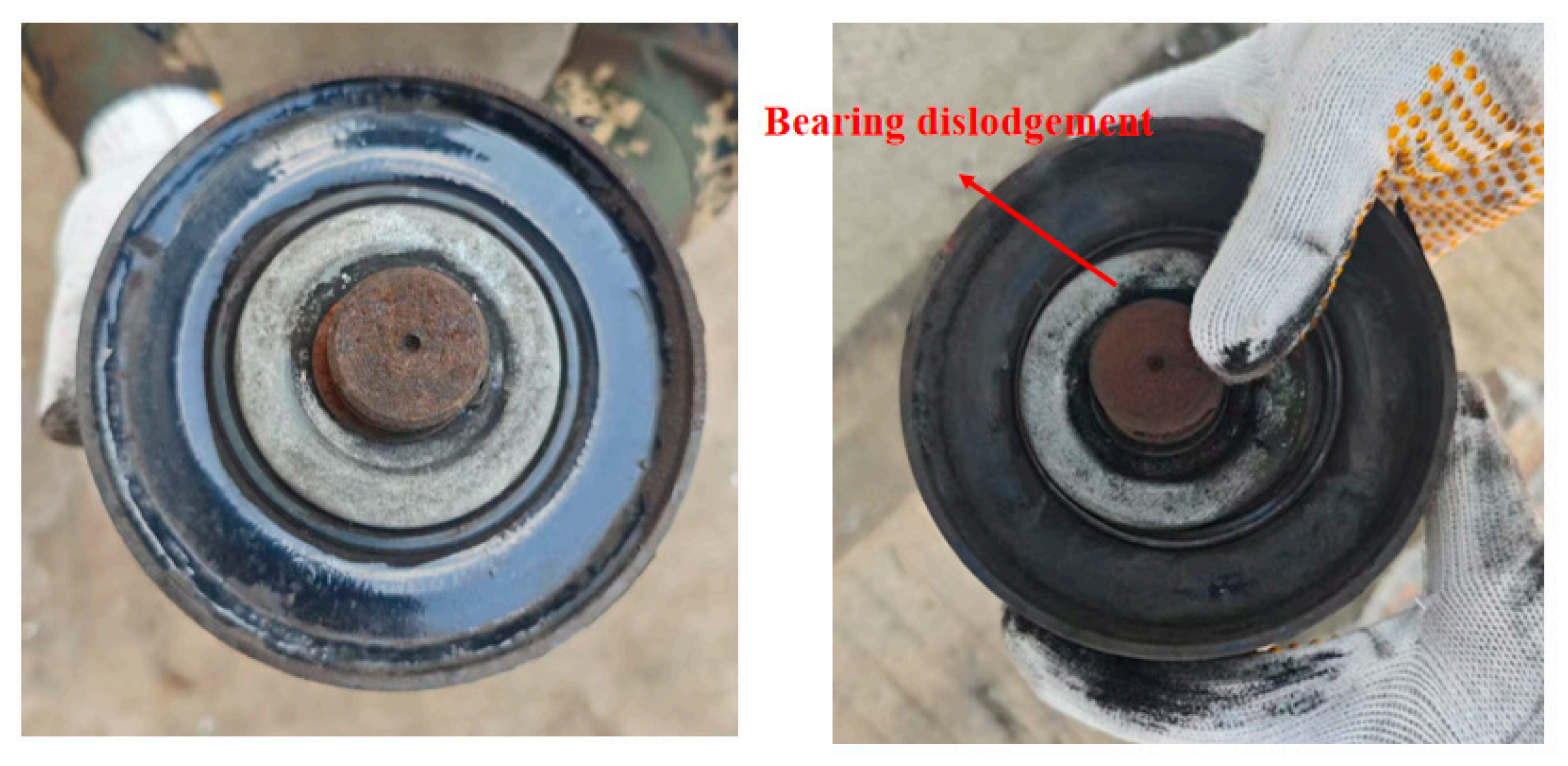
Normal Idler and Bearing-Fault Idler.

To analyze the vibration response differences among various idler fault types and to verify the capability of the UWFBG array-based vibration monitoring system in identifying idlers with different structures, this study conducted comparative experiments on a belt conveyor sample section based on field investigations. Field surveys of the coal handling system in thermal power plants reveal that the upward short idlers are numerous, operate under high loads and high intensity over long periods, resulting in a significantly higher failure rate than the downward long idlers. Common faults of short idlers mainly include bearing detachment and bearing seizure, while long idlers, supporting the unloaded belt and bearing relatively small forces, exhibit a more singular fault pattern, primarily component aging faults such as bearing looseness. Accordingly, three typical fault types were selected for the experiment: bearing detachment of short idlers, bearing seizure of short idlers, and bearing looseness of long idlers. The normal idler and representative bearing-fault idlers are shown in Figure 13.

The fault-type dataset contained 100 samples for each of three classes (300 samples in total). For classification, each signal was mean-removed, filtered by a fourth-order zero-phase Butterworth band-pass filter from 2 to 480 Hz, and converted to its Hilbert envelope. The relative energy gains G_1_, G_2_, and G_3_ were calculated for 0–50 Hz, 100–150 Hz, and 300–350 Hz, respectively, using the normal signal from the same measurement zone as the reference. An additional structural ratio G_h_ was defined from the normalized ratio between the third- and first-band energies. The decision thresholds were defined as follows:

(15) $$T_{1}=\frac{1}{2}\left[\mathrm{median}(G_{1}|A)+\mathrm{median}(G_{1}|B\cup C)\right]$$

(16) $$T_{2}=\frac{1}{2}\left[\mathrm{median}(G_{2}|B)+\mathrm{median}(G_{2}|A\cup C)\right]$$

(17) $$T_{h}=\frac{1}{2}\left[\mathrm{median}(G_{h}|A)+\mathrm{median}(G_{h}|C)\right]+\gamma \left|\mathrm{median}(G_{h}|C)-\mathrm{median}(G_{h}|A)\right|,\;\;\;\;\gamma =0.1$$

**Figure 14:**
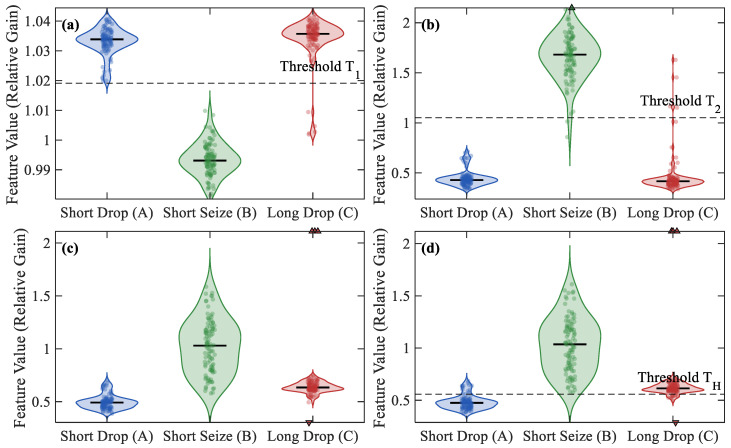
Feature distributions and decision thresholds for the three idler fault types. (**a**) Relative energy gain *G*_1_ in the 0–50 Hz band and threshold *T*_1_; (**b**) relative energy gain *G*_2_ in the 100–150 Hz band and threshold *T*_2_; (**c**) relative energy gain *G*_3_ in the 300–350 Hz band; (**d**) structural ratio *G_h_* and threshold *T_h_*. Triangles indicate samples outside the displayed y-axis range.

A stratified five-fold cross-validation procedure was used to avoid evaluating the classifier on the same samples used to determine the thresholds. In each fold, four subsets were used to calculate T_1_, T_2_, and T_h_, and the resulting thresholds were applied to the held-out subset. The class proportions were preserved in every fold, the random seed was fixed at 42 for reproducibility, and the 10% margin coefficient γ was kept constant across folds to reduce overlap between Classes A and C. For visualization of the complete feature distributions in Figure 14, the thresholds estimated from all samples were T_1_ = 1.0191, T_2_ = 1.0518, and T_h_ = 0.5493; these full-sample thresholds were not used to calculate the cross-validation performance.

To provide a comparison with a conventional time-domain feature, the RMS value was calculated directly from each preprocessed vibration sample as follows:

(18) $$\mathrm{RMS}=\sqrt{\frac{1}{N_{s}}\sum_{n=1}^{N_{s}}x_{s}^{2}(n)},\;\;\;\;n=1,2,\ldots ,N_{s}$$

The RMS comparison used the same 300 samples, preprocessing procedure, and stratified five-fold partitions as the proposed method. In each fold, the median RMS value of each fault class was calculated only from the training subsets, and each held-out sample was assigned to the class with the nearest median RMS value. No additional reference normalization was introduced.

The aggregated five-fold results are listed in Table 1, the comparison with the conventional RMS feature is summarized in Table 2, and Figure 14 shows the feature distributions and the full-sample decision boundaries. The fold accuracies were 95.0%, 90.0%, 83.3%, 95.0%, and 88.3%, giving a mean accuracy of 90.3% with a standard deviation of 4.9 percentage points. Across all held-out predictions, 271 of 300 samples were correctly classified, corresponding to an overall accuracy of 90.3% and a 95% Wilson confidence interval of 86.5–93.2%. For Classes A, B, and C, the precision values were 91.6%, 96.1%, and 86.9%; the recall values were 87.0%, 98.0%, and 86.0%; and the F1-scores were 89.2%, 97.0%, and 86.4%, respectively. The principal confusion occurred between short-idler bearing detachment and long-idler bearing looseness, whereas the short-idler seizure class remained the most readily distinguishable.

The conventional RMS feature yielded a cross-validated accuracy of 80.3%, which was lower than the 90.3% obtained using the proposed characteristic-energy features. RMS retained relatively high sensitivity to Class B, but the lower recalls for Classes A and C indicate that a single overall-amplitude feature cannot fully distinguish faults with similar vibration intensity but different spectral-energy distributions. By incorporating the relative energy gains of multiple frequency bands and the inter-band energy ratio, the proposed method achieved more balanced class-wise performance.

## 4. Conclusions

This study developed a UWFBG-DAS method for long-distance belt-conveyor idler monitoring. Finite-element analysis showed that the middle crossbeam provides an effective vibration-transmission path and exhibits a pronounced low-frequency response, supporting the deployment of the sensing array. Envelope demodulation and VMD were then used to extract the IMF2 frequency-band energy, while temporal accumulation and a zone-specific 95th-percentile threshold enhanced persistent fault information and reduced the influence of structural differences. In the approximately 1.2 km field deployment, characteristic changes were detected in 9 of 10 idler-replacement tests. For the 300-sample fault-type dataset, stratified five-fold cross-validation achieved an overall accuracy of 90.3% (95% confidence interval: 86.5–93.2%). These results demonstrate the feasibility of the proposed method for long-distance idler monitoring under spatially heterogeneous industrial conditions.

The present finite-element model focuses on the principal support components and vibration-transmission paths. Future work will incorporate the belt, detailed bearing contacts, drive drums, and neighboring sections, together with experimental modal validation, to further improve the structural model. The influence of operating speed, load, and sensor coupling on the selection of K and the fault-sensitive IMF will also be investigated. A same-condition comparison with Rayleigh-scattering DAS was not conducted because a synchronized reference system was unavailable during the field campaign. In addition, larger independent datasets and controlled comparisons with Rayleigh-scattering DAS will be used to evaluate the generalization of the method.

## Figures and Tables

**Table 1:** Aggregated classification results from stratified five-fold cross-validation.

Category	Predicted A	Predicted B	Predicted C	Uncertain
A	87	0	13	0
B	0	98	0	2
C	8	4	86	2

**Table 2:** Comparison of the conventional RMS feature and the proposed method.

Method	Accuracy	95% CI	Recall A	Recall B	Recall C	Macro-F1
Conventional RMS	80.3%	75.5–84.4%	78.0%	93.0%	70.0%	80.2%
Proposed method	90.3%	86.5–93.2%	87.0%	98.0%	86.0%	90.9%

## Data Availability

The original contributions presented in this study are included in the article. Further inquiries can be directed to the corresponding author.
